# Detection of Esophageal Squamous Cell Carcinoma by Cathepsin B Activity in Nude Mice

**DOI:** 10.1371/journal.pone.0092351

**Published:** 2014-03-11

**Authors:** Wei Ma, Lie Ma, Hong Zhe, Cihang Bao, Nana Wang, Shaoqi Yang, Kai Wang, Fangli Cao, Yanna Cheng, Yufeng Cheng

**Affiliations:** 1 Department of Radiation Oncology, Qilu Hospital of Shandong University, Jinan, China; 2 Department of Radiation Oncology, Cancer Hospital, General Hospital of Ningxia Medical University, Yinchuan, China; 3 Department of Cardiology, Cardiovascular and Cerebrovascular Disease Hospital, General Hospital of Ningxia Medical University, Yinchuan, China; 4 Digestive System Department, General Hospital of Ningxia Medical University, Yinchuan, China; 5 Department of Oncology, Wendeng Center Hospital, Weihai, China; 6 Department of Oncology, Liaocheng People's Hospital, Liaocheng, China; 7 School of Pharmaceutical Sciences, Shandong University, Jinan, China; The University of Hong Kong, China

## Abstract

**Background and Objective:**

Despite great progress in treatment, the prognosis for patients with esophageal squamous cell carcinoma (ESCC) remains poor, highlighting the importance of early detection. Although upper endoscopy can be used for the screening of esophagus, it has limited sensitivity for early stage disease. Thus, development of new diagnosis approach to improve diagnostic capabilities for early detection of ESCC is an important need. The aim of this study was to assess the feasibility of using cathepsin B (CB) as a novel imaging target for the detection of human ESCC by near-infrared optical imaging in nude mice.

**Methods:**

Initially, we examined specimens from normal human esophageal tissue, intraepithelial neoplasia lesions, tumor in situ, ESCC and two cell lines including one human ESCC cell line (Eca-109) and one normal human esophageal epithelial cell line (HET-1A) for CB expression by immunohistochemistry and western blot, respectively. Next, the ability of a novel CB activatable near-infrared fluorescence (NIRF) probe detecting CB activity presented in Eca-109 cells was confirmed by immunocytochemistry. We also performed *in vivo* imaging of tumor bearing mice injected with the CB probe and *ex vivo* imaging of resected tumor xenografts and visceral organs using a living imaging system. Finally, the sources of fluorescence signals in tumor tissue and CB expression in visceral organs were identified by histology.

**Results:**

CB was absent in normal human esophageal mucosa, but it was overexpressed in ESCC and its precursor lesions. The novel probe for CB activity specifically detected ESCC xenografts *in vivo* and *in vitro*.

**Conclusions:**

CB was highly upregulated in human ESCC and its precursor lesions. The elevated CB expression in ESCC allowed *in vivo* and *in vitro* detection of ESCC xenografts in nude mice. Our results support the usefulness of CB activity as a potential imaging target for the detection of human ESCC.

## Introduction

In China, esophageal cancer ranks fourth in terms of incidence and mortality among all cancers, with an estimated 259,235 new cases and 211,084 deaths in 2008 [1]. While esophageal adenocarcinoma (EAC) has emerged as the major type in some western countries, esophageal squamous cell carcinoma (ESCC) is the predominant type in China [2]–[4]. For ESCC, patients in stages I and II_a_ with negative lymph node metastasis, five-year survival exceeds in 60% after radical surgery. Although the treatments for ESCC have made great progress, the prognosis for patients with advanced disease (II_b_∼IV) still remains poor and unsatisfactory [5], [6], with a five-year survival around 30% [7], [8]. All these facts together clearly indicate the need for a molecular biomarker for ESCC enabling earlier detection.

Although many molecular biomarker candidates of ESCC, such as squamous cell carcinoma antigen (SCC-Ag), carcinoembryonic antigen (CEA), CA19–9, and mutated p53, have been identified; biomarkers with the necessary sensitivity and specificity are still lacking [9]. The most widely utilized blood-based biomarker is SCC-Ag, which is not expressed in all patients. Neither it is highly specific (as it is elevated in other SCC) nor useful for early detection of the disease. Furthermore, SCC-Ag levels neither provide information regarding the localization of the disease nor the existence of metastases. Currently, invasive endoscopy is the most commonly used diagnosis for ESCC; however, it may lead to esophageal injury and easily miss early cancers due to patchy nature of the dysplasia [10].

Cathepsin B (CB) is lysosomal proteolytic enzyme, which is involved in a variety of physiological events including lysosomal protein turnover, angiogenesis, apoptosis, and immune responses as well as pathological events [11]–[15]. CB has been found to be upregulated in several tumor entities, namely in the lung, colorectal, prostate, breast, and melanoma [16]–[20]; however, CB expression in ESCC currently remains unclear. In the current study, we examined CB expression in human ESCC and its precursor lesions and tested the feasibility of using CB as a novel imaging biomarker for the detection of ESCC by near-infrared optical imaging in nude mice *in vivo* and *in vitro*.

## Materials and Methods

### CB expression in esophageal tissues

We retrieved 80 tissue blocks (formalin-fixed, paraffin-embedded) of esophagus biopsy and ESCC resection from pathology department in our hospital. Those tissue specimens included normal tissue (n = 20), intraepithelial neoplasia (IN) lesions (n = 20), tumor in situ (Tis) (n = 20) and ESCC (n = 20). All patients, from whom the tissue specimens were collected, provided their informed consent regarding this study. The protocol was approved by the Ethics Committee of Qilu hospital, Shandong University. (The reason why some consents were verbal: the patients, who live in rural areas far away from our hospital, rarely came back to hospital to get their physical examination done after surgery. In these cases, we could only explain our purpose through phone calls. With patients' permission, the staffs who work in follow-up office of our hospital filled the informed consent form in written and send them to the Ethics Committee. Furthermore, our study is retrospective in nature, and we used only surgical specimens.) Data were anonymized prior to analysis.

The collected tissue blocks were cut into 5 μm-thick sections and mounted on glass slides. Following mounting, the sections were de-paraffinized in xylene and dehydrated in ethanol. Next, the samples were microwaved in 10 mM citrate buffer (pH 6.0) for 20 min to unmask the epitopes and treated with 3% hydrogen peroxide for 10 min. They were finally incubated with primary antibody (1∶50, ABGENT, San Diego, CA) at 4°C for 12 h. After washing, horseradish peroxidase/Fab polymer conjugate (EnVision Plus/HRP peroxidase kit; Dako, Carpinteria, CA) was applied to the sections at 37°C for 30 min. Finally, the sections were incubated with diaminobenzidine for 1 min to develop the signal. Each series included negative control sections incubated without the primary antibody.

### Cell culture

Human ESCC cell line, Eca-109 and normal human esophageal epithelial cell line, HET-1A were obtained from Cell Resource Center, IBMS, CAMS/PUMC, Beijing, China, which were grown in DMEM and RPMI-1640 Medium (Gibco, Life technologies, Bleiswijk, The Netherlands), respectively, supplemented with 10% fetal bovine serum, 50 units/ml of penicillin and 50 µg/ml streptomycin. Cells were maintained in a humidified incubator at 37°C and 5% CO_2_.

### Immunoblot analysis for CB levels in two lines

Eca-109 cells and Het-1A cells were cultured until full confluence in 35 mm culture dishes. Then, the cultured cells were treated with lysis buffer (100 µl/dish) for protein extraction and estimated with BCA protein assay (Beyotime, Jiangsu, China) for protein concentrations. Equivalent amounts of the cell lysates (30 µg) were electrophorezed on reducing 15% SDS-polyacrylamide gels, and the separated proteins were transferred to PVDF membranes. Human CB antibody mentioned above (1∶200) and β-actin (1∶5000, Sigma, St. Louis, MO) were incubated with the membrane at 4°C overnight. After incubating with HRP-conjugated secondary antibodies, the resulting IgGs were detected using the Western Lighting reagent.

### CB probe

The probe used for this study is a commercially available protease activatable near-infrared fluorescence (NIRF) probe, Cat B 680 FAST ™ (PerkinElmer, Inc., Boston, USA). This CB activatable agent is optically silent upon injection and produces fluorescent signal after cleavage by CB.

### Detection of CB activity in Eca-109 cells using the CB probe

Eca-109 cells were seeded at a density of 5×10^4^ cells/well in 24-well cover-glass bottom plates and incubated for 36 h. After initial incubation, the CB probe (200 nM) was added into each well and again incubated for 3 h. Subsequently, the cells were rinsed with PBS and fixed in 4% paraformaldehyde in PBS for 10 min at room temperature (RT). After washing for three times, the cells were permeabilized in 0.5% Triton X-100 in PBS for 5 min at RT. The permeabilized cells were incubated with goat serum for 10 min to block unspecific binding of the antibody, and each well was incubated in the 1∶100 diluted CB antibody (ABGENT, San Diego, CA) for 2 h. A negative control group omitted the primary antibody. The goat anti-rabbit IgG (H+L) antibody, FITC conjugate (1∶100 dilution; Beijing Biosynthesis Biotechnology Co., LTD, Beijing, China) was applied to each well for 30 min in darkness. After three times of wash in PBS, the nuclei of the cells were counterstained with diamidino-2-phenylindole (DAPI). Fluorescence images were obtained with laser scanning confocal microscope (LSCM) with FITC and Cy5.5 filters at 400× magnification (Olympus, Tokyo, Japan).

### Mice model

Five-week old male immunodeficient BALB/c nude mice (n = 30) were obtained from Beijing Unilever Inc., Beijing, China. They were housed at Qilu Hospital animal facility. In order to induce an ESCC model, 1×10^6^ Eca-109 cells suspended in 100 µl sterile PBS were injected subcutaneously into the left flank of 20 mice and allowed to grow for 30 days. Tumor growth was monitored daily. All animal studies were approved by the Ethics Committee of Qilu hospital, Shandong University (Approval No. 12036) and conducted in compliance with European guidelines for the care and use of laboratory animals.

### *In vivo* imaging of tumor bearing mice

When the diameter of tumor xenografts reached 7.0±3.0 mm in size, 2 nmol (100 µl) of the CB probe was injected via tail vein following the probe manual. After injection, the anesthetized mice (n = 6) were placed in the heated imaging platform, the NIRF signals were non-invasively monitored using a living imaging system (IVIS Kinetic, Caliper Life Sciences, USA). Image acquisition parameters were as follows: emission filter  =  Cy5.5, excitation filter  = 675 nm, emission spectra  = 680 to 720 nm, exposure time  = 0.5 s, f/stop  = 2, binning  =  medium, field of view  = 12.5 cm^2^. *In vivo* imaging of tumor bearing mice were performed every 2 h, 36 h post the CB probe injection, and acquired images were analyzed using living image software (version 4.2.0, Caliper Life Sciences, Inc., US). Fluorescence intensity, defined as total radiant efficiency [p/s]/[μW/cm^2^], was quantified using identical size regions of interest (ROI). The tumor ROIs were placed with the center over the respective brightest fluorescence in the tumor xenografts. For consistency, the same investigator placed all ROIs. The data were shown as mean ± SD. Normal nude mice (n = 3) acted as control group.

### *Ex vivo* imaging of resected tumor xenografts and visceral organs

To further assess the NIRF signal intensity in the body, six tumor bearing mice were sacrificed and perfused with normal saline at 30 hrs after injecting the CB probe when the fluorescence signals in tumor sites showed peak intensity *in vivo*. Then, tumor xenografts and main visceral organs including liver, kidneys, heart, lung, esophagus, thyroid gland, testes, spleen, and intestine were excised, rinsed with PBS and imaged with the living imaging system. Image acquisition parameters were same as *in vivo* imaging. Acquired images were analyzed using the living image software. Fluorescence intensity, defined as average radiant efficiency [p/s/cm^2^/sr]/[μW/cm^2^], was quantified using various size ROIs which illustrated the outlines of different organs. The data were shown as mean ± SD. Normal visceral organs (n = 3) acted as control group.

### NIRF histology of resected tumor xenografts

To verify the sources of fluorescence signals in tumor xenografts imaged *in vivo* and *in vitro*, the tumor xenografts were snap frozen and made into cryosections at the end of *ex vivo* imaging. The sections were counterstained with DAPI and observed with a fluorescence microscopy with Cy5.5 filter (BX51, Olympus, Japan).

### CB expression in tumor xenografts and visceral organs

To understand CB presence in different tissues, after *ex vivo* imaging of resected tumors and visceral organs, they were fixed in 4% formalin, embedded in paraffin, and cut into 5 μm-thick sections for immnohistochemistry (IHC). The steps were same as described above and the primary antibody was mouse CB antibody (1∶20, R&D Systems, Inc., Minneapolis, USA). The paraffin sections were observed with a light microscopy (BX51, Olympus, Japan).

### Statistical analysis

Statistically significant differences were determined by two tailed Independent-Sample Student's t test (*p*<0.05 was taken as significant) using statistical software SPSS 16 (SPSS Inc., Chicago, IL).

## Results

### Overexpression of CB in ESCC and its precursor lesions

**Figure 1:**
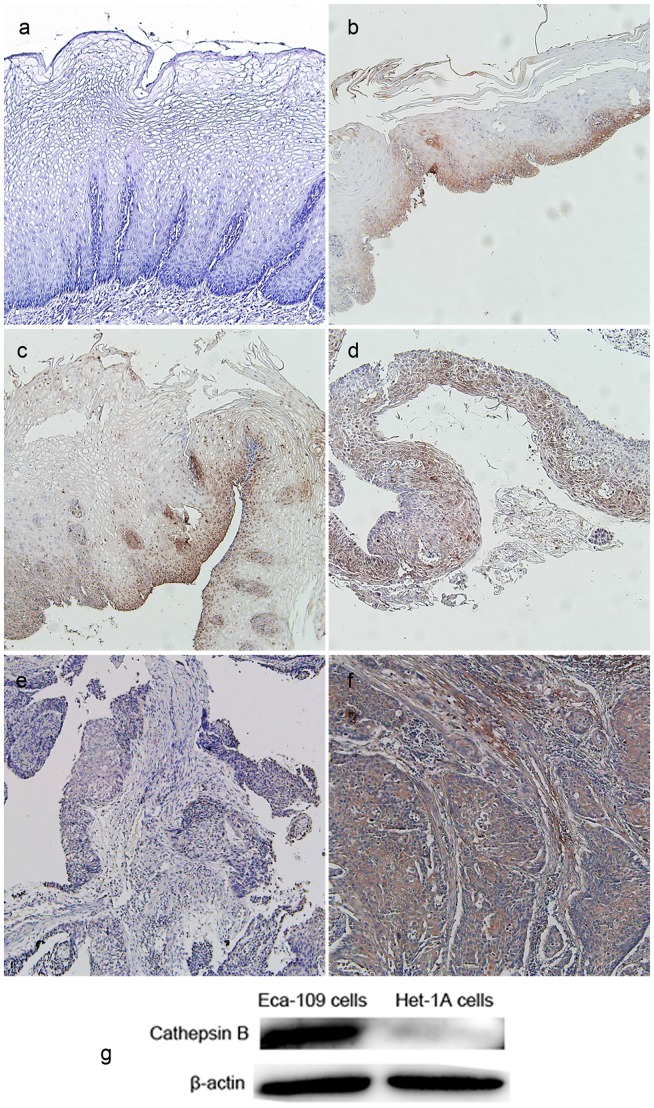
Cathepsin B (CB) expression in human esophageal tissues and two cell lines. Positive expression was observed in intraepithelial neoplasia (IN) I(b), IN II(c), IN III (d), tumor in situ (e) and esophageal squamous cell carcinoma (f) but not in normal esophageal mucosa (a). (g) Eca-109 cells (human esophageal squamous cell carcinoma cell line) had a high level of CB expression, but Het-1A cells (normal human esophageal epithelial cell line) had no CB expression. β-actin was used as an internal control. Original magnification, 100×.

CB has been previously reported to be involved in the invasion of esophageal cancer [21], [22]. We further confirmed CB expression in human ESCC including its precursor lesions by IHC. We observed that CB was absent in normal esophageal mucosa. In contrast, it was found to be highly upregulated in IN (I∼III), Tis and ESCC (Fig. 1a–f). The percentages of CB positive expression in normal mucosa, IN lesions, Tis and ESCC were 0 (0/20), 95% (19/20), 95% (19/20) and 100% (20/20), respectively. CB was mainly expressed in the cytoplasm of cancer cells or epithelial cells. The results suggest the potential of CB to serve as an early biomarker of ESCC. In addition, while immunoblot analysis revealed a high level of CB expression in Eca-109 tumor cells, normal esophageal intraepithelial cells (Het-1A cells) had no CB expression (Fig. 1g), which was in accordance with IHC results.

### Detection of CB activity in tumor cells with the CB probe

**Figure 2:**
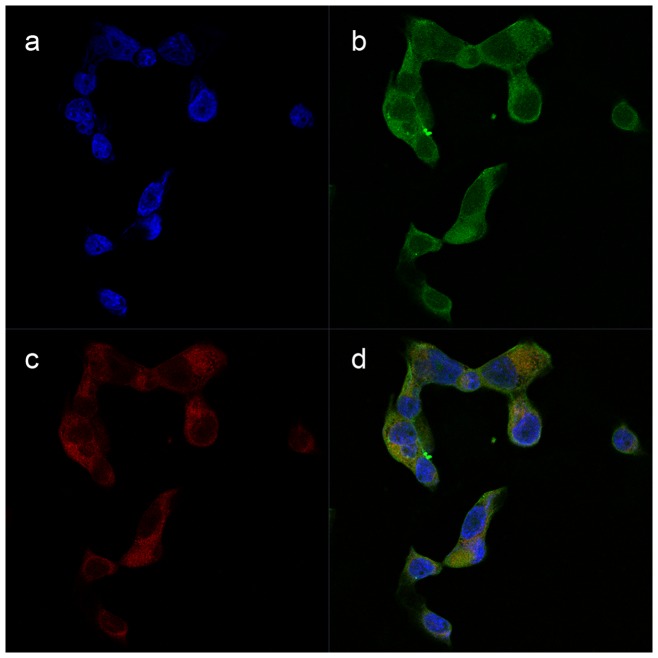
Detection of cathepsin B (CB) activity in Eca-109 cells by the CB probe. (a) Nuclei of Eca-109 cells which were stained with DAPI (Blue). (b) CB expression in cytoplasm of Eca-109 cells staining with FITC (Green). (c) Fluorescence signals in cytoplasm of Eca-109 cells due to the probe activation (Red). (d) The complete coincidence of CB expression and fluorescence signals in merged image indicating the high specificity of the CB probe to CB activity. Original magnification, 400×.

IHC image, in Fig. 1f, displayed diffuse cytoplasmic expression of CB in ESCC entity. High CB expression was also observed in cytoplasm of Eca-109 tumor cells (Fig. 2b). In response to CB activity, the recovered NIRF signals of the CB probe were limited in cytoplasm of Eca-109 cells (Fig. 2c) and completely coincided with CB distribution (Fig. 2d). This indicates that the probe molecules were first taken in by Eca-109 tumor cells, next met with specific CB, and finally generated fluorescence signal after CB degradation. Therefore, in cultured Eca-109 tumor cells, NIRF signal recovery of the CB probe was closely related with CB expression. No significant CB positive signals were observed in negative control.

### Selective detection of tumor xenografs *in vivo* with the CB probe

**Figure 3:**
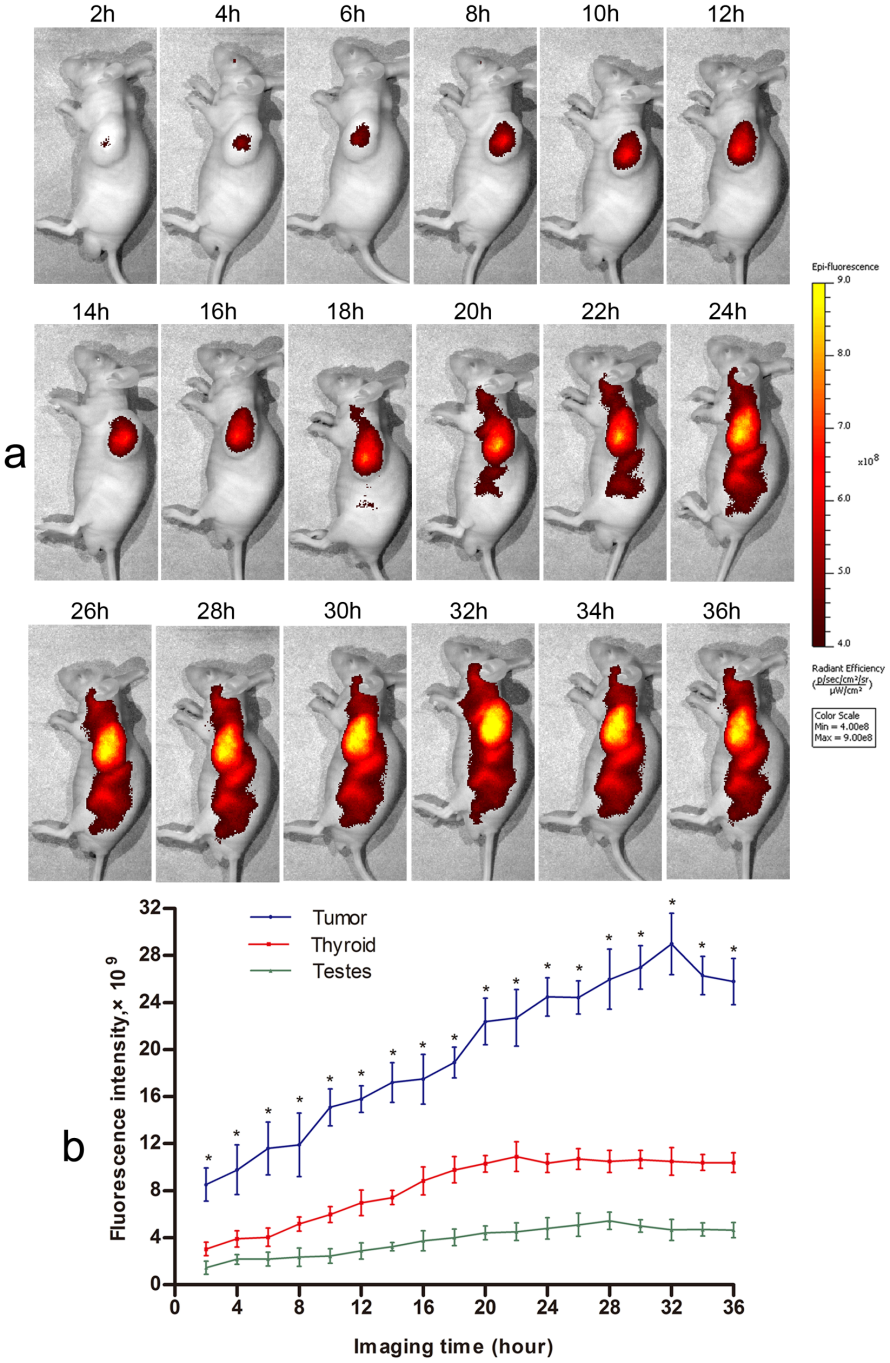
Representative *in vivo* imaging of tumor bearing mouse after injection with the cathepsin B probe (n = 6). (a) A strong specific fluorescence signal in tumor site for 36 hrs. (b) Comparison of fluorescence intensities in tumor site and thyroid/testis at each time point (^*^ *p*<0.05 when compared with thyroid and testis). Error bars represent SD.

Six tumor bearing mice were imaged for 36 h after injecting the CB activatable NIRF probe. The whole mice body initially emitted weak homogeneous autofluorescence (1.34×10^5^±3.3×10^4^), and there were no significant differences in mean fluorescence intensity (FI) between tumor sites and other parts of the body. Two hours after injecting the probe, we observed a strong specific fluorescence signal localized at the tumor sites (9.78×10^9^±1.1×10^9^) which gradually increased up to about 32 hrs (2.90×10^10^±1.84×10^9^) in tumor bearing mice compared to normal nude mice (Fig. 3). This indicates that the CB probe could accumulate in tumor tissue, enter into tumor cells, and recover NIRF signals in cytoplasm in response to CB activity. In addition, thyroid gland and testis showed relatively higher fluorescence signal during imaging time, although compared with the FIs at tumor site, their FIs were significantly lower at each imaging time point (all *p*<0.05) and also, the signals kept changing to some extent (Fig. 3b). Fig. 3a shows a sample of *in vivo* imaging acquired from six tumor bearing mice. For the control mice, the left flank areas showed no significant NIRF signals (2.04×10^6^±5.3×10^5^) during whole imaging time; however, their thyroid and testis showed significant signals similar to that tumor bearing mice.

### Selective detection of tumor xenografs *ex vivo* with the CB probe

**Figure 4:**
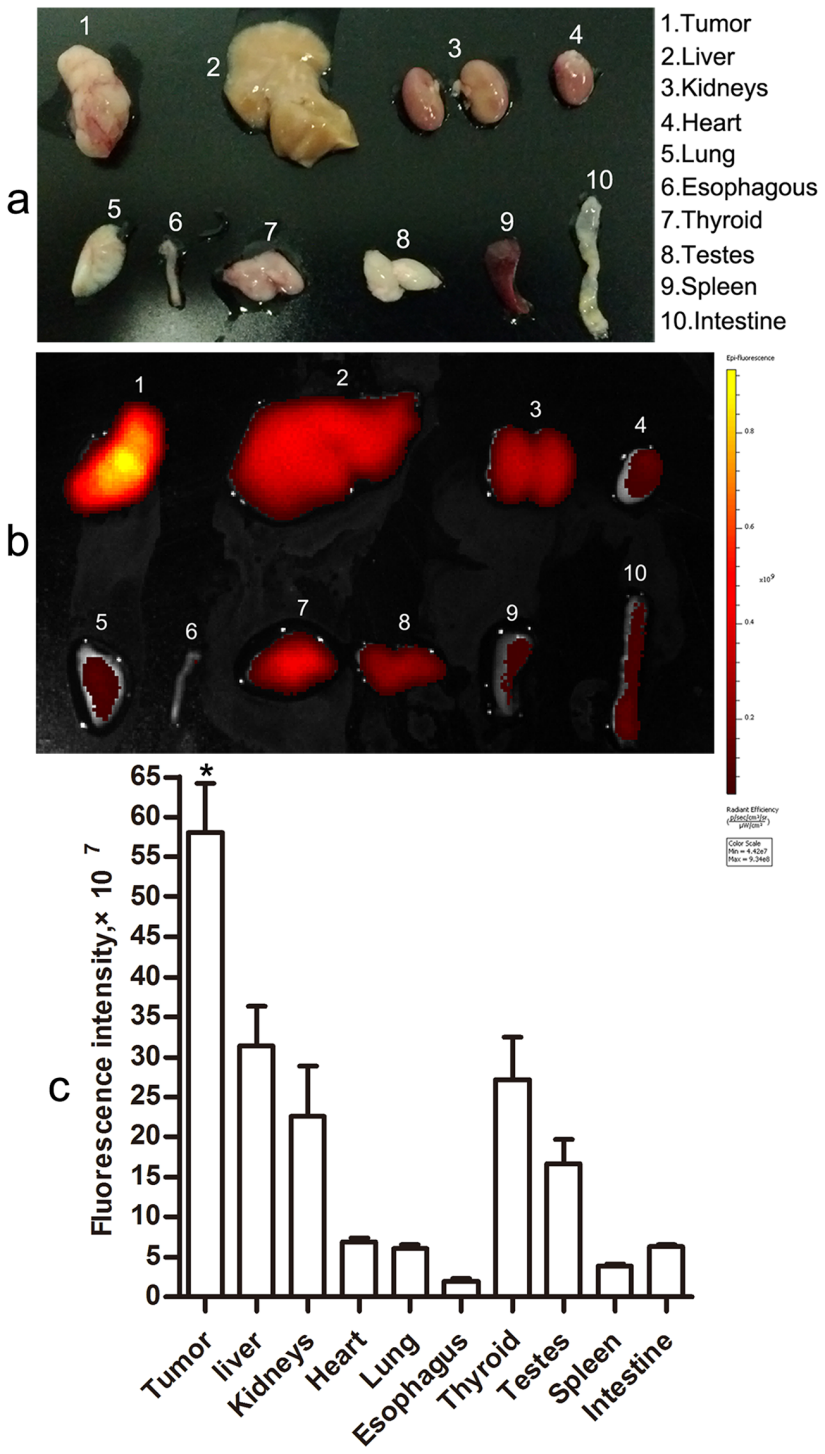
Representative *ex vivo* imaging of the resected tumor xenografts and visceral organs (n = 6). (a) A photograph of the excised tumor and visceral organs using a digital camera. (b) A representative *ex vivo* image of the resected tumor xenografts and visceral organs from tumor bearing mice sacrificed at about 30 hrs after injection with the CB probe. (c) Comparison of fluorescence intensities in tumor site and main visceral organs (^*^ *p*<0.05 when compared with visceral organs). Error bars represent SD.

To compare the signals directly, different tissues were subjected to side-by-side imaging comparisons and quantitative fluorescence signal analysis (Fig. 4). Similar to *in vivo* imaging, tumor xenografts showed the strongest NIRF signals in *ex vivo* imaging (5.81×10^8^±4.3×10^7^) compared to the visceral organs (all *p*<0.05), which reconfirmed tumor specificity of the CB probe. However, notably, the probe displayed relatively higher fluorescence signals in visceral organs including liver, kidney, thyroid gland, and testis (3.14×10^8^±3.5×10^7^, 2.26×10^8^±4.4×10^7^, 2.71×10^8^±3.8×10^7^ and 1.66×10^8^±2.2×10^7^, respectively). The tumor to visceral organs signal ratios were as follows: tumor/liver 1.85, tumor/kidney 2.57, tumor/heart 8.4, tumor/lung 9.57, tumor/esophagus 29.20, tumor/thyroid 2.14, tumor/testis 5.31, tumor/spleen 15.17, and tumor/small intestine 9.21. Among the tumor xenografts and visceral organs, the fluorescence signals of esophagus were lowest (1.99×10^7^±2.5×10^6^). Furthermore, heart and lung which are near to esophagus also showed lower signals.

### Sources of fluorescence signals in tumor xenografs

**Figure 5:**
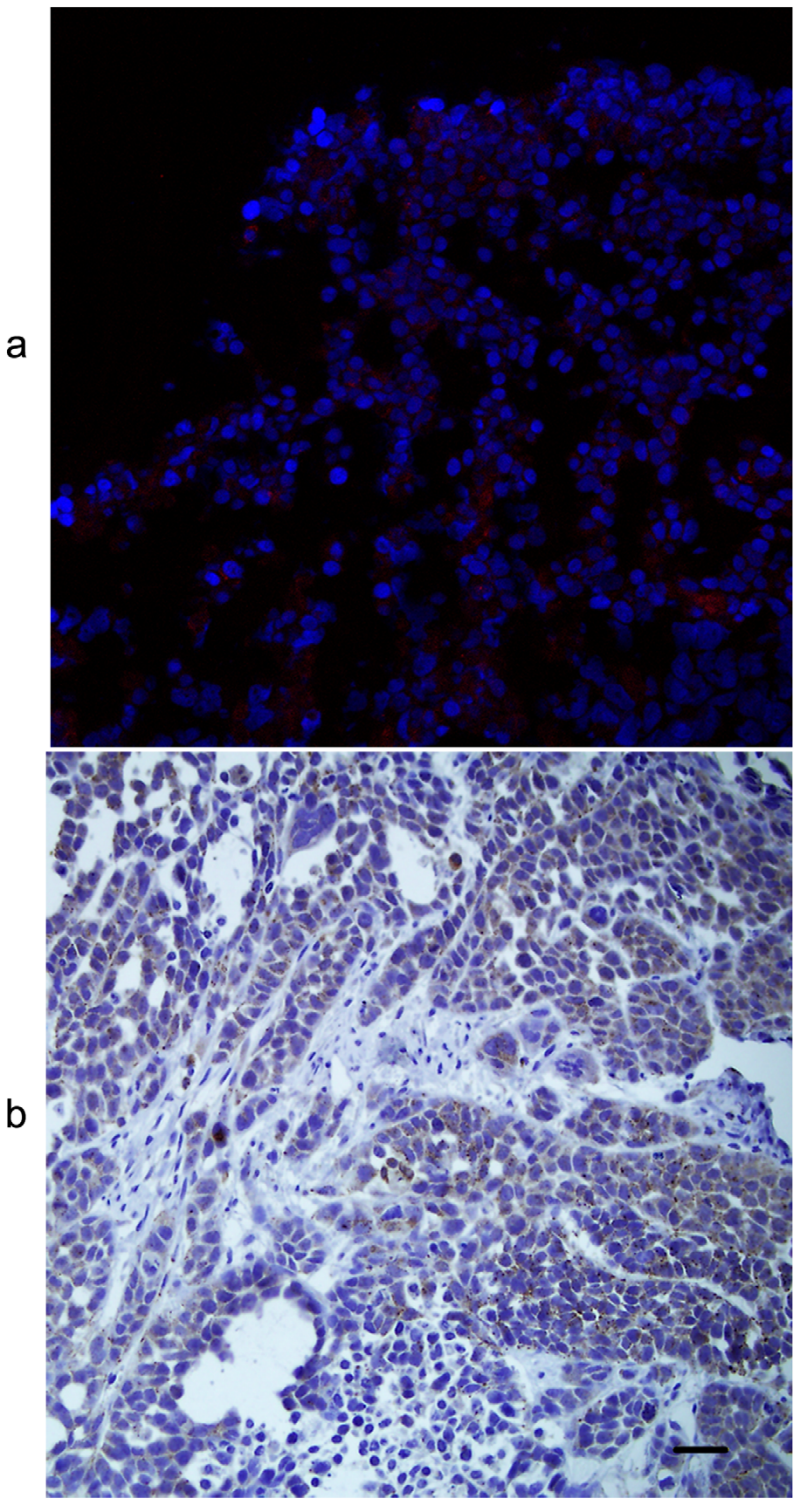
Near-infrared fluorescence (NIRF) histology of the resected tumor xenografts. (a) NIRF signals (red) of the cathepsin B (CB) probe in the cytoplasm of Eca-109 tumor cells counterstained with DAPI (blue). (b) CB expression (brown) in the cytoplasm of Eca-109 tumor cells which coincided with the location of NIRF signals of the CB probe. Original magnification, 200×.

NIRF histology showed that the fluorescence signals in tumor tissue were from cytoplasm of Eca-109 tumor cells (Fig. 5a), which coincided with the location of CB expression (Fig. 5b). The result demonstrated that Eca-109 tumor cells were the sources of fluorescence signals in ESCC xenografts imaged *in vivo* and *ex vivo*.

### Expression of CB in tumor xenografts, liver, kidney, thyroid gland, and testis

**Figure 6:**
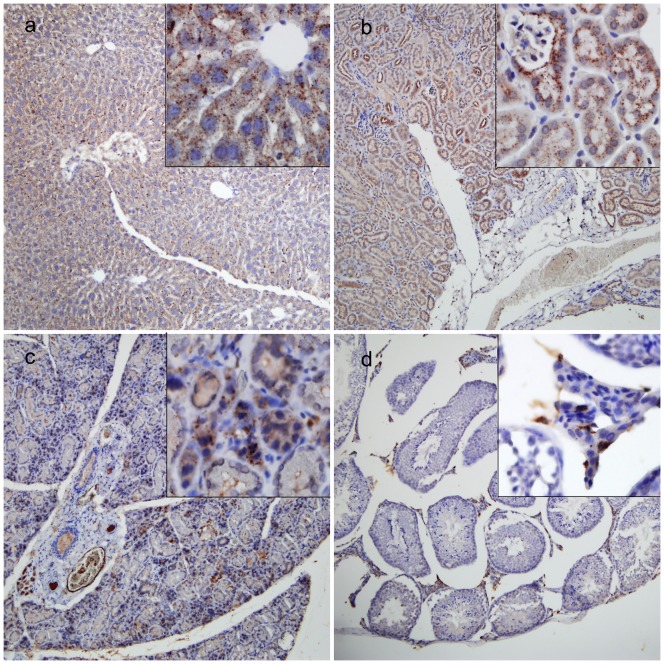
Cathepsin B (CB) expression in some visceral organs showing higher fluorescence signals in Figure 5. CB positive expression (brown) was observed in liver (a), kidney (b), thyroid gland (c) and testis (d) tissues, respectively. Original magnification, 100× and 400× (panels).

The ESCC xenografts of tumor bearing mice developed a prominent stroma similar to that observed in human ESCC, and high CB expression was present in cytoplasm of Eca-109 tumor cells, but not in stromal components (Fig. 5b). To explain the reason why the CB probe detected in some normal organs *in vivo* or *ex vivo*, we examined CB expression in resected internal organs. CB positivity was observed in the cytoplasm of hepatocytes and endothelial cells of hepatic sinusoid, cytoplasm of renal tubular epithelial cells, parafollicular cells and colloids secreted from follicular epithelial cells, and cytoplasm and nuclei of testicular interstitial cells for mouse liver, kidney, thyroid gland, and testis (Fig. 6). Moreover, CB expression levels histologically correlated with the fluorescence signals (Figs. 4, 5, and 6). No CB expression was seen in mouse heart, lung, esophagus, spleen, and intestine (data not shown).

## Discussion

To the best of our knowledge, this is the first report which shows CB overexpression in ESCC and its precursor lesions including IN and Tis. Our results suggest the potential of CB to serve as an early biomarker of ESCC. We described here the effectiveness of selecting CB as an imaging target in detecting ESCC. The results indicate potential of further development in clinically relevant imaging and therapeutic agents based on the enzymatic activity of CB in ESCC.

The novel CB probe used in our study, Cat B 680 FAST, was developed by PerkinElmer in 2011. It is composed of a CB-specific peptide flanked by two near-infrared fluorochromes and a pharmacokinetic modifier for optimal *in vivo* imaging. Upon CB cleavage of the substrate sequence, the agent becomes highly fluorescent in target areas. This architecture offers higher target specific signals with reduced background compared to previous similar probe such as proSense 680 and proSense 750 [23], [24]. ProSense 680 and proSense 750 are broad cathepsins activatable fluorescent imaging probes which are sensitive to cathepsin B/H/L/S instead of single CB. In addition, proSense 680 was also reported to be activated mostly by tumor-associated macrophages, not by tumor cells. Our results also confirmed the sensitivity and specificity of this CB probe in detecting CB activity (Figs. 2–6). This is the first demonstration of a novel NIRF imaging agent specific for CB that can localize and detect ESCC xenografts. Similarly, fluorescent imaging probes in combination with near-infrared optical imaging has been applied to more sensitive detection of a series of cancers including pancreas, hypopharynx, ovary, and lung cancers [24]–[29]. Proteases activatable NIRF probes enabled detection of molecules or biologic activities of tumors in real time [30], [31].

Normal organs such as liver, kidney, thyroid gland, and testis also showed relatively higher fluorescence signals both in the *in vivo* and *ex vivo* imaging in our study. Subsequently, we confirmed CB presence in these organs by IHC. Our findings are in agreement with the previous reports [32], [33]. Obviously, it was the target protease (CB) produced by non-tumor cells in these organs that decreased tumor-specificity of the CB probe. On the contrary, the ability of this probe to detect arbitrary organs in which CB expresses slightly proves that the probe is highly sensitive and specific to CB activity.

It seems that CB target is more suitable for the detection of ESCC with optical imaging *in vivo*. Liver and kidney might have more interference in the diagnosis of orthotopic tumors in abdomen such as pancreatic cancer due to anatomical location. But liver, kidney and thyroid are far away from thoracic esophagus in which the incidence of esophageal cancer is highest. Moreover, our results showed that the organs around esophagus such as heart and lung had very low fluorescence signal (Figure 4), which would not impact the detection of esophagus *in vivo*. In addition, it is technically feasible to detect orthotopic esophageal cancer by a flexible LSCM combined with fluorescence probes.

Our study utilized near-infrared optical imaging as a basic technique to explore the specificity and ability of a novel CB probe to detect ESCC xenografts in mouse model. However, single near-infrared optical imaging may not be the most suitable or the best modality for ESCC detection in the clinic due to fluorescence limited penetration. For hollow organs like esophagus, near-infrared optical imaging combined with endoscopy seems to be a better choice. White light upper endoscopy combined with near-infrared imaging with a CB activatable probe (Prosense750) enhanced the detection of EAC in an orthotopic murine model [24]. Using a cathepsin activatable near-infrared probe in combination with flexible LSCM in a genetically defined mouse model, pancreatic ductal adenocarcinoma and pancreatic intraepithelial neoplasia lesions were detected and graded in real time *in vivo* [25]. Another study demonstrated that capsule endoscopy can be combined with molecular imaging and fluorescence signals of different intensities with adenomas were detected *in vivo* [34]. Moreover, CB target can be adopted to many other imaging modalities or multimodality imaging [35].

In conclusion, high CB expression in ESCC allowed the detection of human ESCC xenograft *in vivo*. Our results indicate the usefulness of CB activity as a potential imaging target for the detection of human ESCC. Although this is only the preliminary work, the noninvasive and specific diagnostic approach for the detection of ESCC is promising. Further studies to find a suitable means for early detection of this deadly cancer will be continued in our research group.
